# Regulation of cellular gene expression by nanomaterials

**DOI:** 10.1186/s40580-018-0166-x

**Published:** 2018-11-30

**Authors:** Sang Hun Chun, Ji Soo Yuk, Soong Ho Um

**Affiliations:** 1 School of Chemical Engineering, Sungkyunkwan University, Suwon, Gyeonggi-do 440-746 South Korea; 2 SKKU Advanced Institute of Nanotechnology (SAINT), Sungkyunkwan University, Suwon, Gyeonggi-do 440-746 South Korea

**Keywords:** Gene regulation, Nanomaterial, Cellular transport, RNA interference

## Abstract

Within a cell there are several mechanisms to regulate gene expression during cellular metabolism, growth, and differentiation. If these do not work properly, the cells will die or develop abnormally and, in some cases, even develop into tumors. Thus, a variety of exogenous and endogenous approaches have been developed that act on essential stages of transcription and translation by affecting the regulation of gene expression in an intended manner. To date, some anticancer strategies have focused on targeting abnormally overexpressed genes termed oncogenes, which have lost the ability to tune gene expression. With the rapid advent of nanotechnology, a few synthetic nanomaterials are being used as gene regulation systems. In many cases, these materials have been employed as nanocarriers to deliver key molecules such as silencing RNAs or antisense oligonucleotides into target cells, but some nanomaterials may be able to effectively modulate gene expression due to their characteristic properties, which include tunable physicochemical properties due to their malleable size and shape. This technology has improved the performance of existing approaches for regulating gene expression and led to the development of new types of advanced regulatory systems. In this short review, we will present some nanomaterials currently used in novel gene regulation systems, focusing on their basic features and practical applications. Based on these findings, it is further envisioned that next-generation gene expression regulation systems involving such nanomaterials will be developed.

## Introduction

The first discovery regarding the regulation of gene expression in natural systems was reported in 1961 by Francois Jacob and Jacques Monod, who studied the Lac operon within *E. coli* [1]. The operon is a unit composed of a structural gene, promoter, and operator. In particular, the Lac operon is a simple unit that regulates the expression of an enzyme involved in lactose metabolism in *E. coli*. Gene regulation inside the cell usually maintains the level of the respective product at a proper concentration and primarily plays an important role in overall cellular metabolism, such as preventing extraneous gene expression or controlling cell growth, proliferation, and differentiation [2–4].

Proper expression of gene products involves regulation of each stage in the central dogma of molecular biology [5]. During the transcription process, regulation is mainly achieved by suppressing the selective and obligatory affinity of RNA polymerases for template DNA. Regulation can also occur through increasing the number of DNA supercoils and thus decreasing the amount of exposed template DNA [6]. In addition to physical regulation by induced supercoiling of DNAs, DNA methylation has been widely proposed to have a permanent regulatory effect through modification of the natural DNA structure, thus blocking the selective binding of some transcriptional factors [7]. The Lac operon is acknowledged to be a representative system for the genetic regulation of template DNAs engaged with RNA polymerases during the course of gene transcription. Most regulatory procedures in the translation process take place at the initiation stage where mRNAs produced through the earlier transcription process are grabbed and read thoroughly by a functional ribosome that is essentially a protein-producing factory. As with the transcription process, translational gene regulation occurs by thwarting the accidental encounter of mRNAs and ribosomes [8]. RNA interference, which is simply abbreviated as RNAi, has been revealed as a natural defense mechanism in plants that suppresses the expression of genes from some foreign bacteria or viruses at the level of translation [9].

Gene regulation has a variety of purposes. For example, genes expressed in the Lac operon are incorporated into template DNAs in a competitive manner with the corresponding RNA polymerase such that the concentration of lactose species is properly maintained in the cell. The MYC family of regulatory genes encodes transcription factors involved in cell growth, proliferation, and differentiation. Especially, for a gene that regulates cell growth such as MYC, dysregulated expression can trigger some life-threatening diseases such as cancer [10]. MYC is one of the best-known oncogenes and is currently being considered as a target gene for both the treatment and diagnosis of cancer [11]. Furthermore, gene dysregulation can lead to several other conditions such as autoimmune disease, inflammation, and obesity [12–14].

In normal cells regulatory mechanisms of gene expression usually include suppression of the expression of abnormal genes, but if this mechanism does not work properly it has to be intentionally regulated with the help of introduced exogenous factors, thus preventing the possible disease outcomes described above. To realize this goal, some studies have attempted to imitate a natural regulatory system in cells. For example, transcriptional inhibition can be achieved by inducing DNA methylation or supercoiling via exogenous inoculation of certain substances [15]. In the case of translation inhibition, a variety of methods that inhibit the activation of translation factors have been proposed [16].

**Fig. 1:**
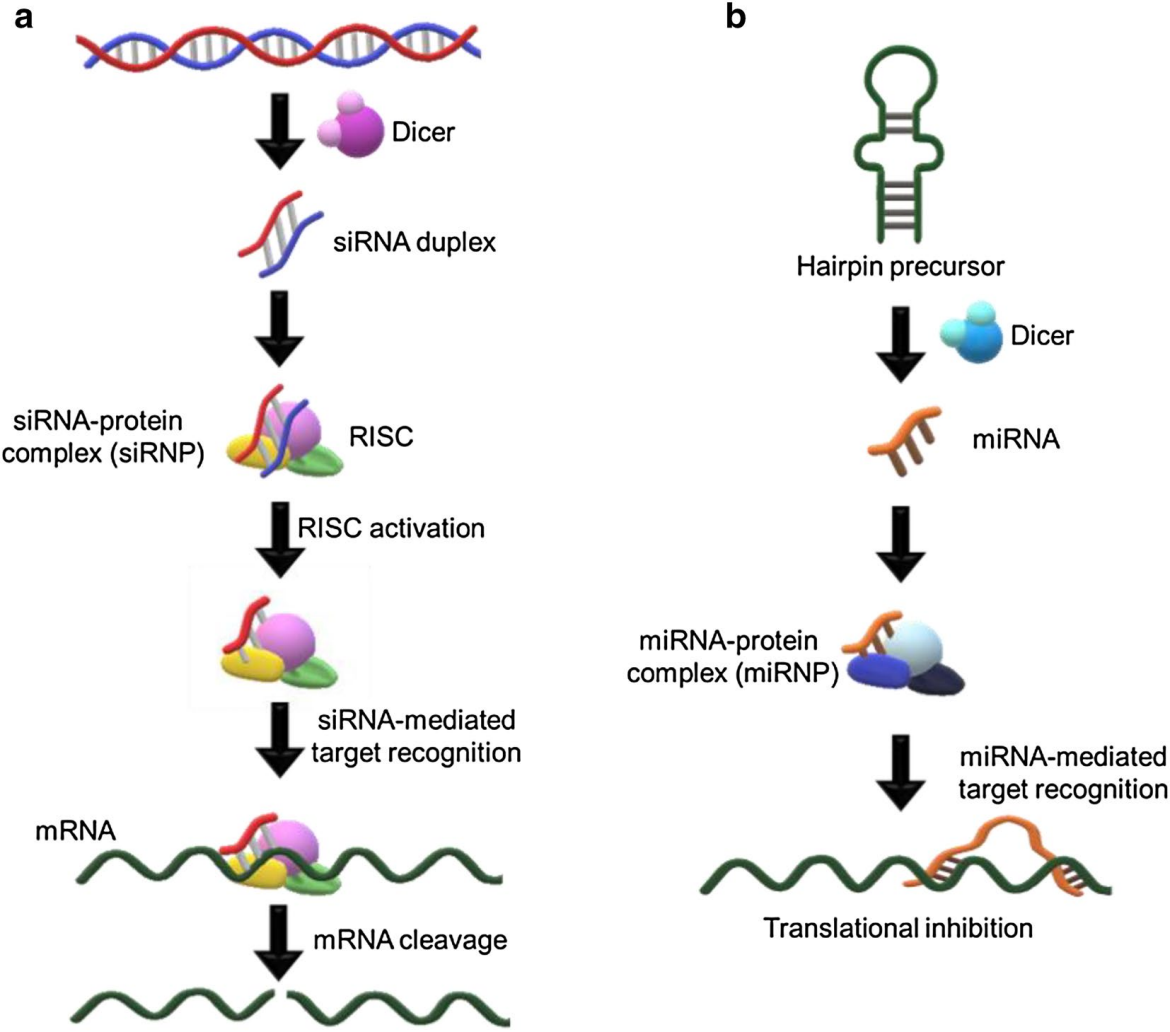
Schematic drawing showing the mechanism of RNAi-based gene regulation: **a** siRNA pathway; **b** miRNA pathway

To date, a novel RNA-mediated regulatory system has been reported as an interesting method of directing the cellular metabolic process [17, 18]. Antisense RNA specifically interacts with target mRNA fragments via complementary hybridization, ultimately accomplishing gene silencing [19]. Notably, since the remarkable discovery of the RNAi mechanism by Andrew Fire and Craig C. Mello in 1998, much progress has been made in the regulation of cellular gene expression by inducing the activity of double-strand RNA (dsRNA) such as miRNA and siRNA for RNAi [20]. Briefly, a long dsRNA or its hairpin precursor has been transformed into shorter strands such as siRNA or miRNA through the enzymatic activity of RNaseIII (i.e., DICER) in a complex. Such RNA-protein complexes, called a RNA-inducing silencing complex (RISC), are able to specifically interact with target mRNAs, leading to transcriptional inhibition in several ways (Fig. 1) [21]. RNAi-based technology is recognized to have much higher selectivity compared with other methods of gene regulation. It thereby reduces the possible off-target effects that can frequently occur and cause many problems for direct clinical applications. As a result of the high efficiency, RNAi is confirmed to have a significant gene knockdown effect even with smaller numbers of effector molecules [22]. Because of these several advantages, RNAi-based gene regulation has become particularly notable in many practical therapeutic applications, as shown in Table 1 [23].

**Table 1:** siRNA-based therapeutics in clinical trials. Reproduced with permission [23]

Start–end date	Drug	Target	Route	Condition	Phase	Clinical trial ID	Sponsors
2010–2012	ALN-TTR01	TTR	IV	TTR-mediated amyloidosis	I	NCT01148953	Alnylam Pharma
2012–2012 2012–2014 2013–2017 2013–2017	Patisiran (ALN-TTR02)	TTR	IV	TTR-mediated amyloidosis	I II II III	NCT01559077 NCT01617967 NCT01961921 NCT01960348	Alnylam Pharma
2013–2015 2013–2015 2014–2017	Revusiran (ALN-TTRSC)	TTR	SC	TTR-cardiac amyloidosis	I II II	NCT01814839 NCT01981837 NCT02292186	Alnylam Pharma
2011–2013 2015–2017	siG12D LODER	KRAS G12D	EUS biopsy needle	Pancreatic ductal adenocarcinoma	I II	NCT01188785 NCT01676259	Silenseed Ltd.
2011–2012 2012–2014	SYL1001	TRPV1	Ophth.	Ocular pain Dryeye syndrome	I I/II	NCT01438281 NCT01776658	Sylentis, S.A.
2011–2012	TKM-080301 (TKM-PLK1)	Polo-kinase-1	IV	Solid tumors with liver involvement	I	NCT01437007	National Cancer Inst.
2010–2014 2014–2016	TKM-080301 (TKM-PLK1)	Polo-kinase-1	IV	Neuroendocrine tumors, adrenocortical carcinoma Hepatocellular carcinoma	I/II I/II	NCT01262235 NCT02191878	Tekmira Pharma
2011–2012	ALN-PCS02	PCSK9	IV	Elevated LDL-cholesterol (LDL-C)	I	NCT01437059	Alnylam Pharma
2014–2015	ALN-PCSSC	PCSK9	SC	Elevated LDL-cholesterol (LDL-C)	I	NCT02314442	Alnylam Pharma
2012	TKM-100201 (TKM-Ebola)	ZEBOV L poly m., VP24, VP35	IV	Ebola virus infection	I	NCT01518881	Tekmira Pharma
2013–2014	ND-L02-s0201	HSP47	IV	Healthy	I	NCT01858935	Nitto Denko Corp.
2014–2016	ND-L02-s0201	HSP47	IV	Moderate to extensive hepatic fibrosis	I	NCT02227459	Nitto Denko Corp.
2014–2015	ALN-AT3Sc	AT	SC	Hemophilia A, hemophilia B	I	NCT02035605	Alnylam Pharma
2014–2016	APN401	E3 ubiquitin ligase Cbl-b	IV	Melanoma, pancreatic cancer, renal cell cancer	I	NCT02166255	Wake Forest Univ.
2014–2016	DCR-MYC	MYC	IV	Hepatocellular carcinoma	I/II	NCT02314052	Dicerna Pharma
2014–2015	DCR-MYC	MYC	IV	Solid tumors, multiple myeloma, non-Hodgkin lymphoma	I	NCT02110563	Dicerna Pharma
2015	siRNA-EphA2-DOPC	EphA2	IV	Advanced cancer	I	NCT01591356	MD Anderson Cancer Center

*AT* antithrombin, *EUS* endoscopic ultrasound, *IV* intravenous, *Ophth.* ophthalmic administration, *PCSK9* proprotein convertase subtilisin/kexin type 9, *TTR* transthyretin, *VP24* viral protein 24, *VP35* viral protein 35, *ZEBOV L polym.* ZEBOV L polymerase

With the rapid advancement of nanotechnology, a variety of nanomaterials that possess unique and excellent physicochemical properties not previously observed have been developed. Many have been exploited for practical and clinical uses in several biomedicine fields. For instance, the intrinsic characteristics of these materials are useful for the design of efficient therapeutic, diagnostic, and imaging agents [24–27]. In this short review, we focus more on progress and studies regarding nanomaterial-based cellular metabolic regulation in more detail, especially on gene regulatory systems based on newly designed nanomaterials.

## Nanomaterials with biological relevance

**Fig. 2:**
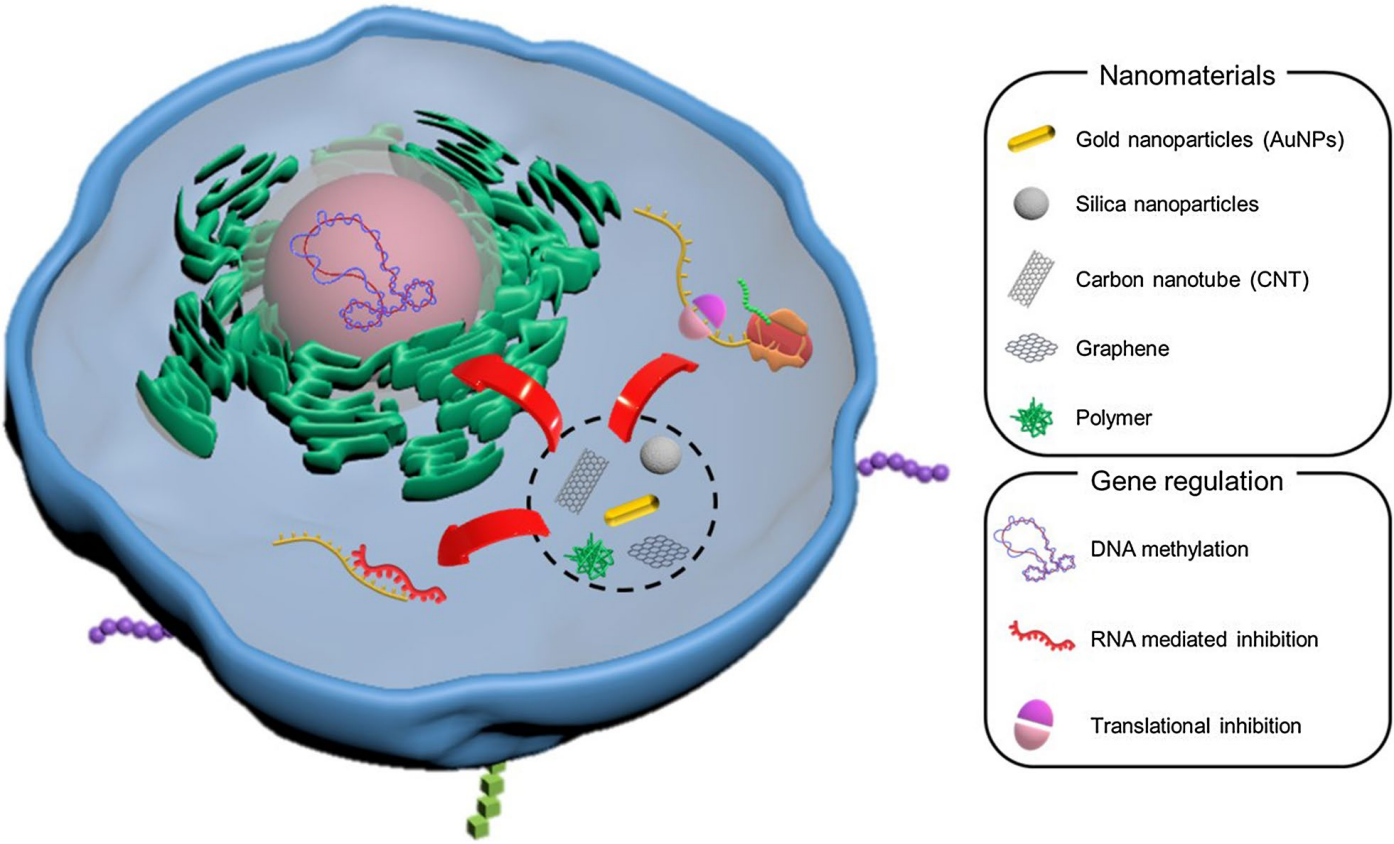
Gene regulation by nanomaterials in a cell

For gene regulations, a variety of nanomaterials have been recently introduced. These materials include carbon and a polymer-based material. Owing to the unique properties of these nanomaterials, they are getting more attention to controllable gene expressions. In principle, polymer-based materials are relatively biocompatible and have lower toxicity compared with other materials, making them available for the uses [28]. Especially, natural polymeric materials derived from living organisms are very attractive due to their intrinsic and easy decomposition in biological conditions [29]. These characteristic features make nanomaterials useful throughout a variety of biomedical fields involving the regulation of cell growth or death, effective delivery of metabolic regulatory medications with specific effects, or enhancing the performance of imaging with high-resolution cellular tracking [30, 31]. Many concerns have to be carefully considered to avoid an immune response induced by exogenous nanomaterials while achieving cellular metabolic regulation with high efficiency [32]. The design of a novel nanomaterial that could overcome the above drawback while maintaining the superior characteristics of intracellular gene regulation is an attractive goal (Fig. 2).

In this review, we cover some representative nanomaterials including general nanoparticles, carbon-based materials, and polymer structures, concentrating on their characteristics and advantages and followed by addressing their limitations and providing perspectives for improvement in their gene-regulatory clinical applications.

### General nanoparticles

The term nanoparticle commonly refers to an organic or inorganic particle with a size of 1–100 nm. Due to the rapid development of nanotechnology, nanoparticles can be easily manufactured with various sizes and shapes and even tailored with some functionalities for eventual use in several fields including medicine and food engineering [33–35]. Intracellular delivery of antagonist RNAs for gene regulation by RNA interference has mostly been achieved with the help of nanoparticle carriers. The surface of nanoparticles is pretreated with functional components that specifically recognize target cells and aid its intracellular entry while avoiding an immune response in the body [36].

Gold nanoparticles, which are simply abbreviated AuNPs, are widely used in bio-medical applications and have been demonstrated to have higher biocompatibility and lower cytotoxicity. Remarkably, AuNPs possess high absorbance at the wavelength of a specific visible light region and exhibit a photothermal effect with generation of heat when irradiated. Because of their unique optical properties they are widely used in both diagnostic and therapeutic systems [37]. AuNPs can easily form a covalent bond with thiol derivatives and can therefore become tightly anchored with some genetic fragments without any complex modification processes [38].

**Fig. 3:**
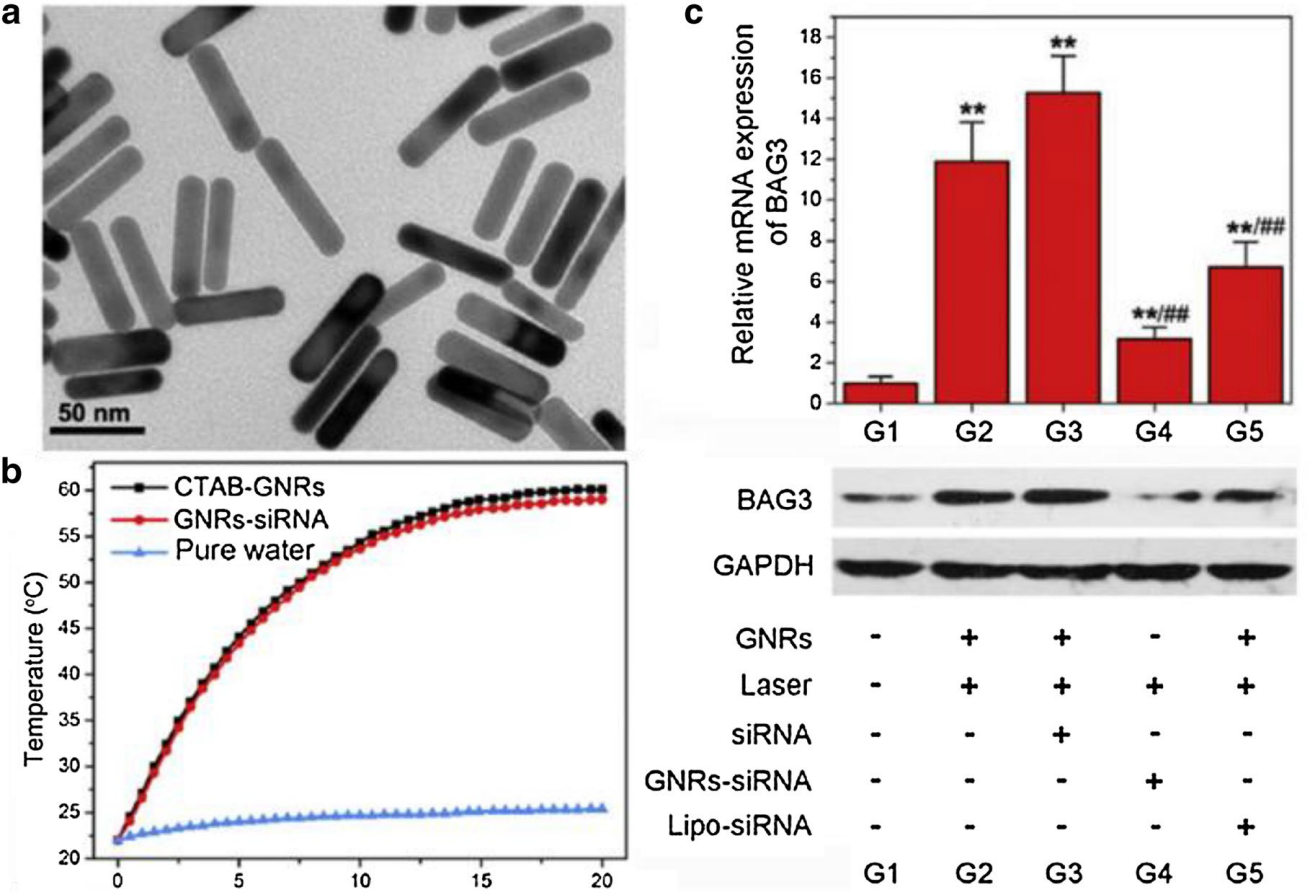
**a** TEM image of GNRs-siRNAs as synthesized and **b** temperature changes after laser irradiation. **c** Changes in the expression level of mRNA encoding BAG3 induced by gold nanorods (Reproduced with permission [39])

Some cancer cells may be highly resistant to external stresses such as heat and drugs. Consequently, it is difficult to achieve sufficient healing effects by simple photothermal therapy or chemotherapy. In a recent study, gold nanoparticles were used as a subsidiary material to maximize the efficacy of photothermal therapy (PTT) through gene regulation. Wang and his colleagues [39] developed a system that enhances the PTT effect of gold nanorods (GNRs) by silencing the *BAG3* gene using siRNA. Here, BAG3 is primarily involved in increasing the heat shock response of cancer cells (Fig. 3). GNRs combined with polymer have shown a higher siRNA transport rate compared with Lipofectamine 2000, which is a commonly used commercially available transfection agent. A sufficient silencing effect was achieved with 30% less siRNA compared to use of Lipofectamine 2000. By suppressing the expression of *BAG3* and reducing the heat shock response of cancer cells, it was possible to achieve a substantial therapeutic effect with low laser power of 600 J/cm, which is much lower than the amount commonly used. As a result, therapeutic treatments were much effective than with PTT alone [39].

Although AuNPs are directly used as a carrier for siRNA delivery, several studies have reported RNAi after a sustained and long-term release of siRNAs adhered onto the surface of nanoparticles by heat generated from AuNPs [40, 41]. As another example, AuNPs incorporating antisense sequences complementary to target mRNAs are internalized into cells, thus depleting mRNAs in the target cells [42]. The reduced level of mRNAs was confirmed by tracking changes in emitted fluorescence. The data confirmed that the system could distinguish mRNAs very sensitively up to a single base difference and 92% of the target mRNAs could be removed [42].

**Fig. 4:**
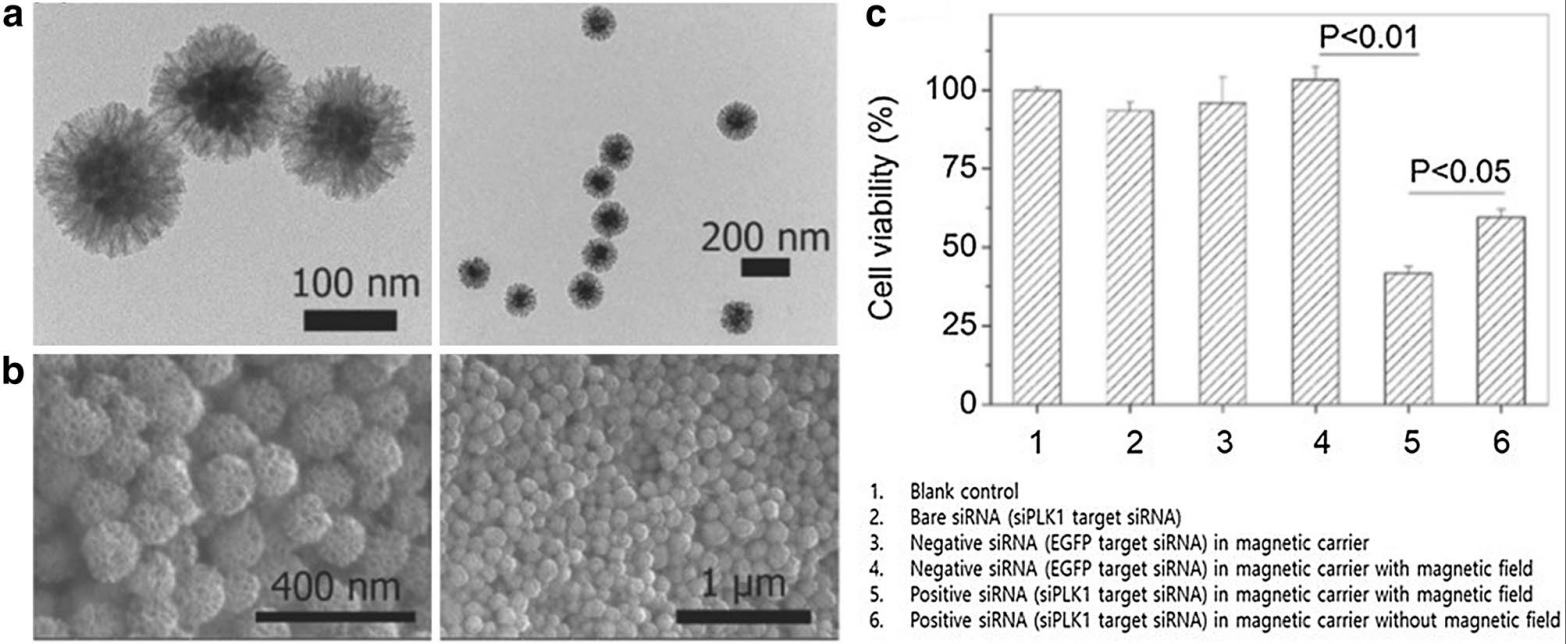
**a** TEM and **b** SEM images of a siRNA carrier made of magnetic particles coated with mesopourous silica. **c** Cell viability after release of siRNAs (Reproduced with permission [45])

Besides AuNPs, magnetic nanoparticles, or MNPs, are widely used as another tool for gene regulation. MNPs including iron, nickel, or cobalt possess a unique superparamagnetic property when in nano-scaled size. Superparamagnetic nanoparticles are not affected in the absence of a magnetic field, but are very sensitive in the presence of external magnetic fields. MNPs have been used to separate and detect some substances in response to external magnetic fields, deliver drugs to targeted areas, and as contrast agents for MRI imaging [43, 44]. According to several recent studies, MNPs can effectively deliver drug molecules for gene regulation in cells. Xiong and colleagues [45] have developed siRNAs-enveloped IONPs with a mesoporous silica layer. These particles could load a large number of siRNAs (up to 2 wt%) as a result of the large surface area (411 m^2^/g) and pore volume (1.13 cm^2^/g) of mesoporous silica. The delicate magnetic characteristics of iron oxide allow localization of more than 40% of particles in cells. As a result, siRNAs transported into human osteosarcoma KHOS cells were successfully guided by an external magnetic field and effectively silenced the target oncogene *PLK1*, resulting in a greater than 60% rate of cell apoptosis (Fig. 4) [45]. Besides AuNPs and MNPs, a variety of nanoparticles such as up-conversion nanoparticles, quantum dots, and silica nanoparticles have also been used in gene regulation systems of siRNA or antisense RNA interference [46–48].

**Fig. 5:**
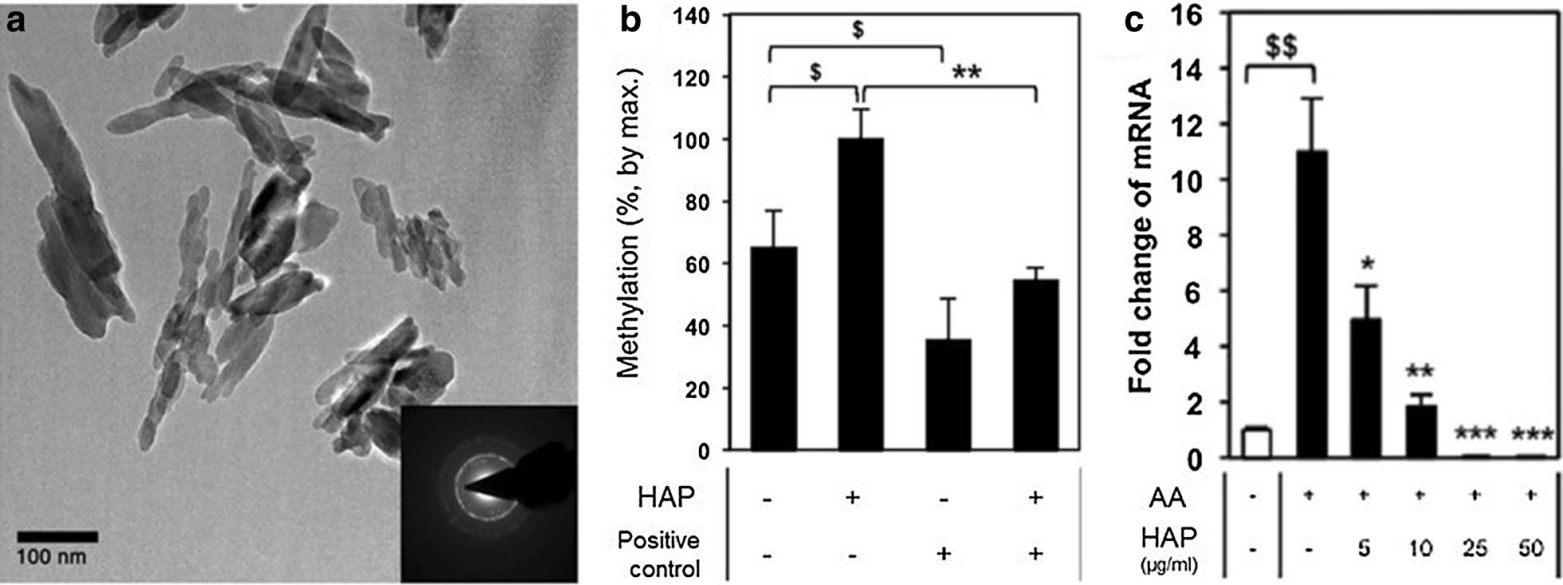
**a** TEM image of synthesized HAPs, **b** methylation ratio of BMSC, and **c** suppression of ALP-related mRNAs according to the number of injected HAPs. The inset of TEM image is electron diffraction patterns by TEM (Reproduced with permission [49])

In addition to RNA interference by nanoparticles, another new method using nanosized hydroxyapatites to induce DNA methylation has been also investigated [49]. Hydroxyapatite (HA) is a naturally occurring mineral consisting of calcium apatite that accounts for 70 wt% of human bones. When synthesized HA (HAP) was injected into bone marrow stromal cells (BMSC), the expression level of key markers and genes relevant to cell differentiation such as alkaline phosphatase (ALP), bone sialoprotein (BSP), and osteopontin (OPN), was significantly decreased according to the amount of HAP injected. In particular, cell differentiation was permanently inhibited at the early stage in HAP-treated cells. The sudden decline in gene expression may be attributed to the promotion of DNA methylation by injected HAPs (Fig. 5) [49].

### Carbon-based materials

Single- and multi-walled carbon nanotubes, graphene, and carbon dots are the most commonly used carbon-based nanomaterials. Because of their higher biocompatibility and lower toxicity relative to other metal materials, they are suitable for several biomedical applications. In particular, they have been used in bioimaging or diagnostic applications due to their unique optical properties [50, 51]. Like AuNPs, they can emit heat through exposure to light radiation.

Furthermore, a ring structure of some carbon-based materials can easily form a non-covalent bond similar to a π–π stacking interaction between genes and carbon molecules. As a result of their high loading efficiency and simple loading process, they are able to effectively transfer genes into cells.

**Fig. 6:**
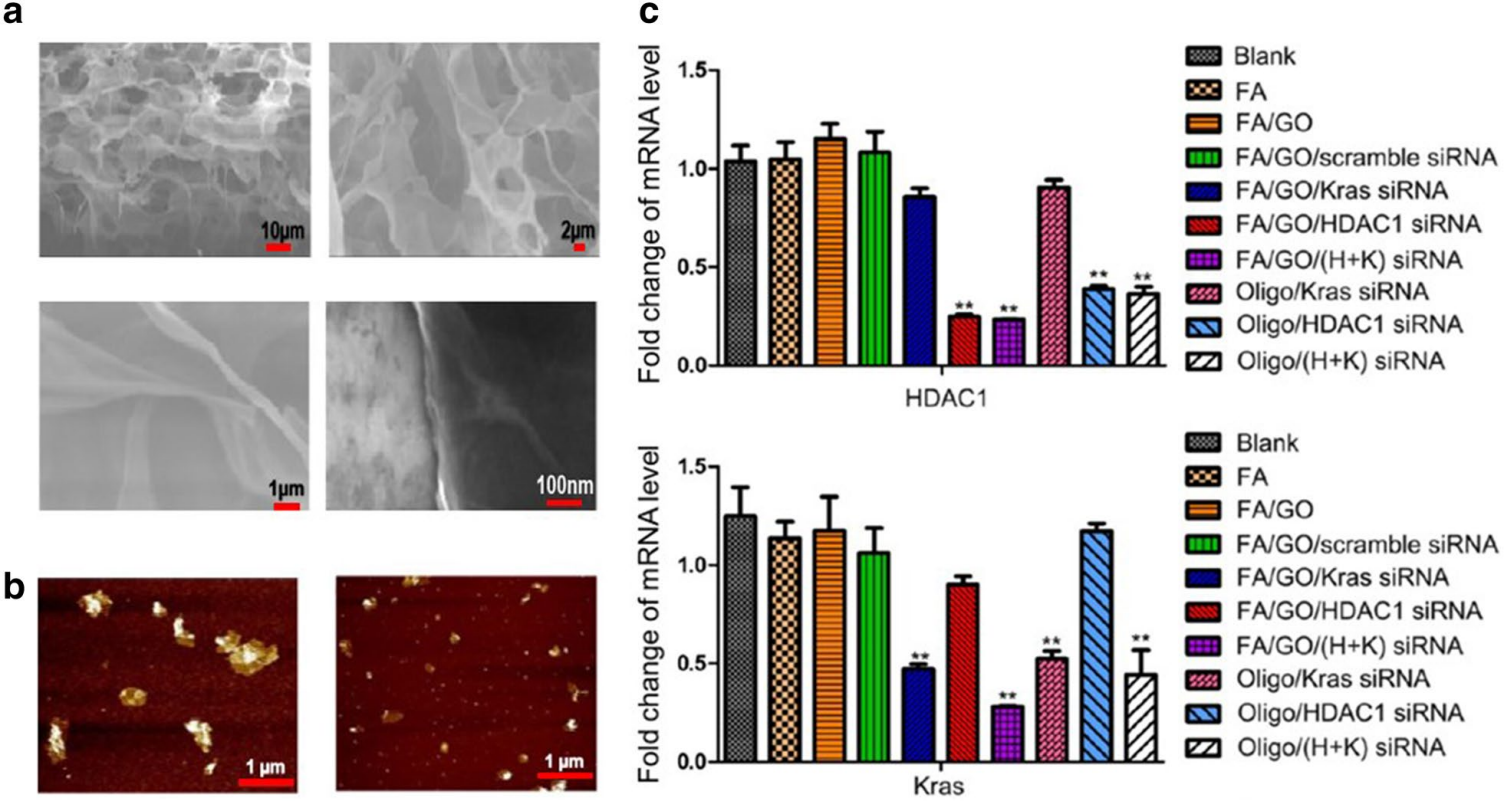
**a** SEM and **b** AFM images of GOx. **c** Regulated mRNA level of targeted HDAC1 and Kras genes (Reproduced with permission [52])

Yin and colleagues [49] have developed PEG- and folic acid (AA)-modified graphene oxide (GO) containing siRNA molecules. When siRNAs were internalized into pancreatic cancer cells using modified GO derivatives, the transfection efficiency was confirmed to be over 90%, and two representative genes essential for cell growth, HDAC1 and K-Ras, were silenced simultaneously. In addition, as a result of the photothermal properties of graphene, more than 80% of cancer cells were removed by a synergetic effect (Fig. 6) [52].

**Fig. 7:**
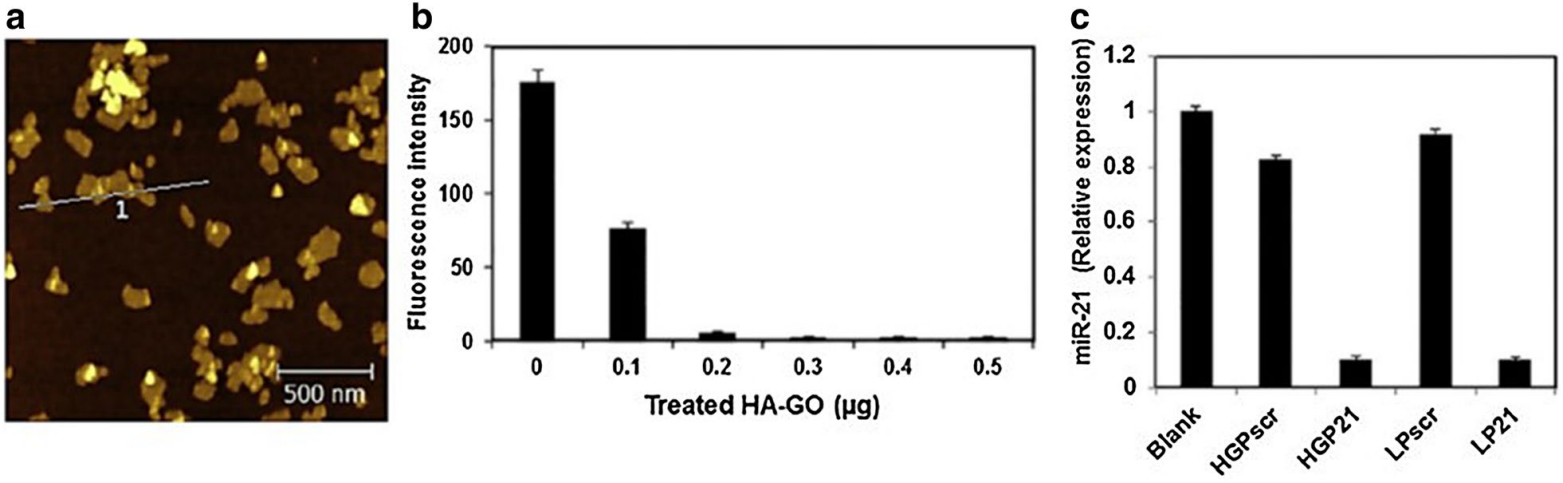
**a** AFM image of synthesized HA-GO (hyaluronic acid–graphene oxide complex) and fluorescence measurement to confirm **b** loading of PNA and **c** targeted miR-21 knockdown. *HG* HA-GO, *L* lipofectamine 2000, *P*_*21*_ PNA for miR-21, *P*_*scr*_ PNA scrambl (Reproduced with permission [53])

An alternative gene regulation system using graphene has been developed by Hwang and colleagues [53]. Knockdown of miR-21 was effectively achieved by delivering peptide nucleic acid (PNA) containing antisense specific for miR21, while simultaneously detecting the target miR-21 and utilizing the quenching effect of graphene. Specifically, it was observed that when fluorescent dyes were attached to PNAs, the fluorescence was quenched immediately when PNAs were very close to graphene. This confirmed the presence of loaded PNAs. Since the PNA and miR-21 in targeted breast cancers were complementary to each other, the fluorescence was quenched and then recovered and miR-21 levels could be detected as soon as PNA was introduced into the targeted cells. Knockdown of miR-21 inhibited the proliferation and migration of cancer cells, eventually inducing their apoptosis (Fig. 7) [53].

As in the previous example, a siRNA delivery system using graphene simultaneously detected the silencing of targeted genes as well as the efficiency of delivery of siRNAs through changes in fluorescence signals due to the quenching ability of graphene. Several similar systems have been developed [54].

**Fig. 8:**
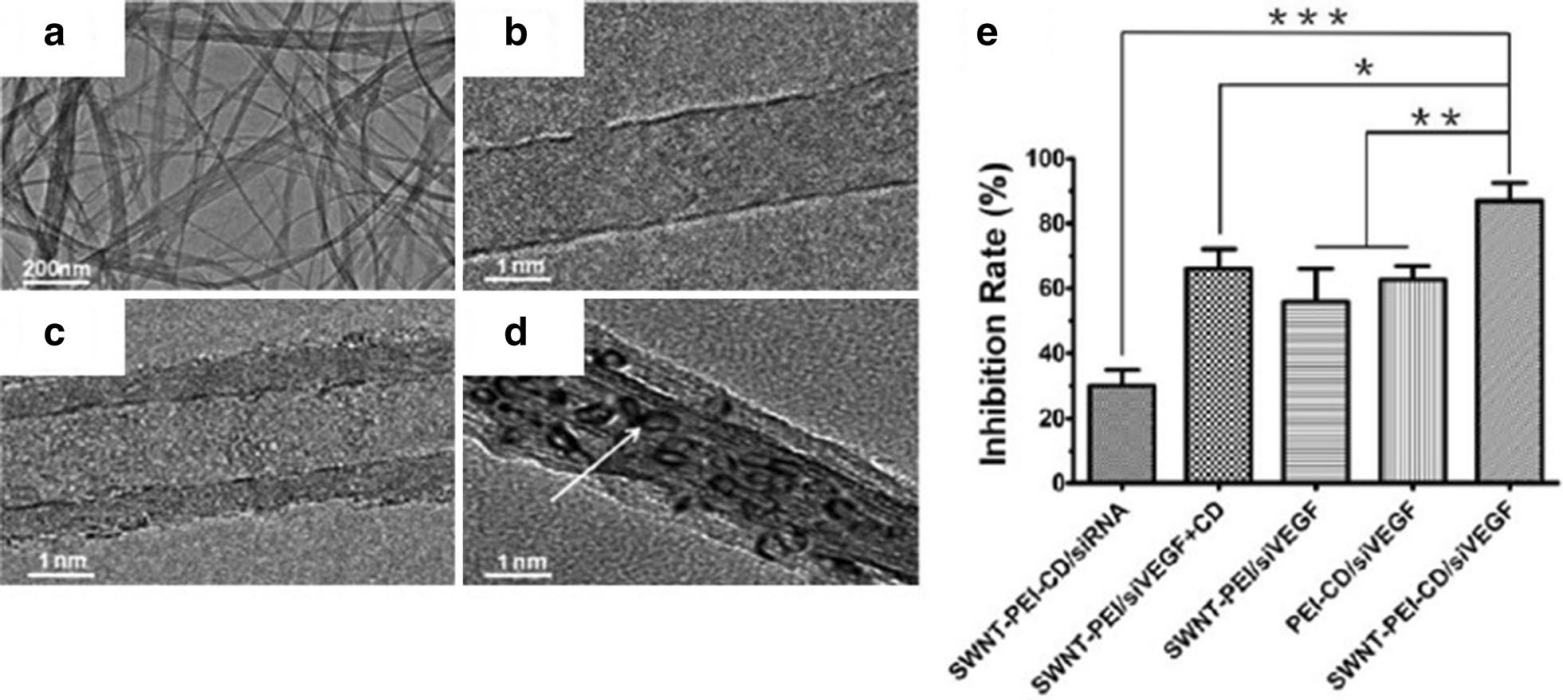
TEM images of **a** purified SWNTs, **b** SWNT − COOH, **c** SWNT− PEI− CD, and **d** SWNT− PEI− CD/siVEGF and **e** regulation of VEGF expression (Reproduced with permission [56])

Carbon nanotubes (CNTs) are other outstanding carbon nanomaterials for gene regulation. CNT is a tubular form with very large surface area. Its high transfection efficiency makes it very advantageous for gene delivery [55]. Ding and colleagues [56] have developed a new siRNA carrier made of polyethylenimine (PEI)-modified single-walled carbon nanotubes (SWNTs). Candesartan (CD) was attached to the surface to target vascular endothelial growth factor (VEGF), which is known to play a significant role in angiogenesis by promoting the formation of new blood vessels. Because angiogenesis is indispensable for the growth of cancer cells, tumor growth can be suppressed by simply inhibiting the expression of VEGF alone. siRNA targeting the VEGF gene was transported into cancer cells through the PEI-SWNTs, resulting in approximately 80% inhibition of VEGF expression in vitro and in vivo (Fig. 8) [56].

### Polymer-based materials

A polymer is a macromolecule in which one or more monomers are repeatedly associated. Polymers possess a wide variety of physical, chemical, electrical, and even optical properties depending on the characteristics of their constituent monomers, their dimensions, and synthesis environment, and can therefore be applied in nearly all academic and industrial fields. Polymers for biomedical uses are pretreated with other ingredients to change their surface charge or to impart specific functional groups. In many cases, polymers have high biocompatibility in the whole system, reducing their toxicity. They are also protected from immune responses or plasma degradation, which enhances their transfection efficiency into targeted cells [57].

In addition, polymers can be filled with certain drugs or effectors through chemical strategies of either covalent attachment or electrostatic attraction [58–60]. In recent years, biopolymers from the body and related synthetic polymer groups have been considered. Most interestingly, polysaccharide-based natural polymers represented by a chitosan and a hyaluronic acid are increasingly being focused on with respect to gene regulation. Zhou and colleagues [61] have synthesized nanoparticles composed of both hyaluronic acid and calcium phosphate. These nanoparticles carried siRNAs targeting Bcl2 genes and aided their cellular transport into melanoma. As a result, they silenced 85% of the targeted genes in vitro and even in vivo, resulting in dramatic apoptosis of targeted cancer cells. Another example of gene regulation using natural polymers has been developed by Yang and colleagues [62]. A basic unit of the system used was a PCSK9 gene regulatory unit based on chitosan oligosaccharides (COS), a polymeric structure composed of *N*-acetyl-d-glucosamine and deacetylated glucosamine.

**Fig. 9:**
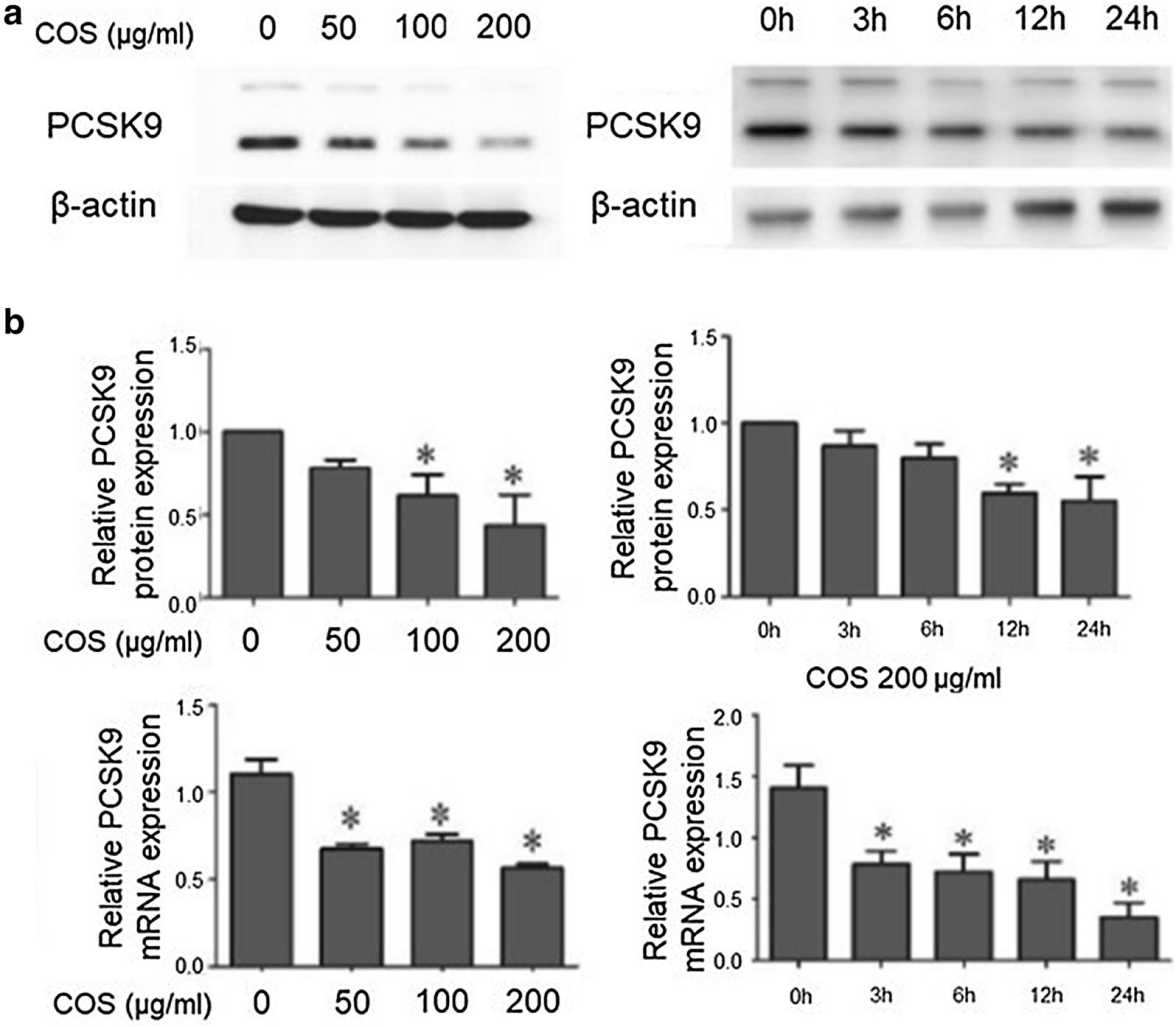
COS downregulates expression of the *PCSK9* gene in HepG2 cells. **a** Western blot result showing expression of PCSK9 protein and **b** RT-PCR analysis of expressed *PCSK9* mRNA according to the number of COS and the time after COS inoculation (Reproduced with permission [62])

COS is known to have antibacterial, anticancer, antioxidant, and anti-inflammatory features. PCSK9 is an enzyme involved in regulation of the biological level of low-density lipoprotein (LDL) in blood vessels. Overexpression of PCSK9 could cause cardiovascular disease by blocking the absorption of LDL. Some relevant studies have demonstrated a mechanism by which cellular COS could promote the formation of a substance that selectively binds to the promoter of the *PCSK9* gene and thereby inhibits PCSK9 expression, eventually allowing the normal removal of LDL in blood vessels (Fig. 9) [62].

## Conclusion and perspective

Cells possess mechanisms to control cellular gene expression and avert unpredicted expression of exogenous genes, thus maintaining normal metabolism. During the transcription process, the expression of gene transcripts is strictly regulated by DNA methylation, acetylation, or deacetylation. Such effects are also controlled by a variety of factors involved in transcription and translation processes. However, when the regulatory mechanism functions abnormally, cells may die or develop into malignant tumors through anomalous proliferation. In such cases, alternative gene regulatory strategies may be required. A method of inducing DNA methylation throughout the transcription process could be proposed. For the translation process, a method of obstructing the binding between mRNA and ribosome by interference with antisense RNAs could be envisioned. Since the discovery of RNAi by siRNA and miRNA, there have been many attempts to realize gene regulation by RNAi. As a result of their tremendous success, most recently developed gene regulation methods have been based on RNAi.

In addition, a variety of nanomaterials have been highlighted. With the rapid development of nanotechnology, several attempts to manipulate nanomaterials into functional moieties have been made to attain effective gene regulation. Most commonly, nanomaterials are used as a better vehicle to efficiently transport effector molecules for either DNA methylation or RNAi to targeted sites in the body. Several nanomaterials including inorganic and organic nanoparticles, carbon-based materials, and polymers have been explored. Relevant studies have mostly focused on efficient delivery of siRNA and antisense RNA for cancer treatment. The intrinsic physicochemical, electrical, and optical properties of the developed nanomaterials result in a higher transfection efficiency, yielding an efficient gene silencing effect.

In this review, we have briefly presented the most recent progress in gene regulation systems using advanced nanomaterials. In most cases, nanomaterials have been used as vehicles to deliver regulatory effectors such as siRNAs, although in some cases they have been directly applied for targeted gene regulation. Current gene regulation systems involving nanomaterials are predominantly focused on RNAi. However, RNAi has low variability and can only be designed for a single target mRNA, leading to incomplete knockdown in the whole system. In addition, siRNA is very vulnerable to external environments and is readily degraded within a short time. As a result, it requires a carrier or must be amended for protection. The need for a transfection agent may eventually reduce feasibility by greatly increasing the net cost of the system. To overcome these shortcomings, a novel gene regulation system is essential for better performance. Nanomaterial-based gene regulatory systems could represent an ideal alternative approach.

In the design of new gene regulatory materials, the following characteristics must be considered. First, either high target selectivity or universal availability is required. One of the reasons that RNA-based gene regulation methods such as siRNA or antisense RNA are attracting attention is because targeted mRNAs specifically react through complementary base hybridization. This not only improves the whole system efficiency by reducing possible off-target gene regulation, but also helps to reduce any side effects such as damage of exogenous DNAs. In addition, it is necessary to regulate many genes at the same time through only one single system. This will be an important characteristic for commercialization of the system by increasing the application in whole-system gene regulation while reducing the total price.

A number of studies have focused on AuNPs and CNTs, which are the most commonly used nanomaterials. However, these materials may cause random DNA methylation and catastrophic damage to targeted cells and genes [63, 64]. Non-specific toxicity to some normal cells and genes has been considered a critical drawback in biomedical applications, including gene regulation. In recent years, numerous efforts to overcome these problems have been attempted through surface modification, size variation, or combination with other materials. For effective and reliable regulation with acceptable safety, the non-specific toxicity of the nanomaterials must be addressed in ingenious ways.

The next generation of gene regulation systems must possess higher regulation efficiency. The RNA-based gene regulation systems developed to date have shown very effective silencing at the level of around 80–90% but have a relatively lower suppressive effect on mRNA compared with the corresponding protein expression. This may be ascribed to the effects of RNAi on the translation process; however, it results in incomplete removal of all mRNAs. Therefore, complete regulation at the transcriptional stage is required for effective gene regulation. This may require a more delicate design strategy for eukaryotic gene regulation because the nanomaterials must enter the nucleus where transcription occurs.

Gene regulatory effectors have evolved from a variety of smaller molecules to antisense RNA and RNAi molecules. There has also been significant progress in terms of transfection efficiency, gene regulation level, and duration. Currently, RNAi-based gene regulation using siRNA is predominantly used, but there are still many drawbacks that must be overcome as described above. Nanomaterials have unique physico-chemical properties, and it is relatively easy to manipulate their size, shape, and surface properties. Therefore, a gene regulation strategy using nanomaterials may be an answer to the next generation of upgraded gene regulation systems that complement the advantages of existing methods. To this end, it is necessary to overcome the above-mentioned disadvantages while moving forward with optimizing the required characteristics.
